# Osteopathic Medical Students’ Concerns About the Future of Abortion Education and Training Post-Roe v. Wade

**DOI:** 10.7759/cureus.60030

**Published:** 2024-05-10

**Authors:** Patricia Zielinski, Nicole Vilar, Tara Lewandowski, Gabriela E Llerena, Sepideh Nadery, Robin J Jacobs

**Affiliations:** 1 College of Medicine, Nova Southeastern University Dr. Kiran C. Patel College of Osteopathic Medicine, Fort Lauderdale, USA

**Keywords:** women’s rights, dobbs decision, women’s health, curriculum, reproductive health, medical education and training, abortion

## Abstract

Background

In the United States, new regulations on access to abortion and, in some cases, penalization of physicians who provide abortions, have been established on a state-by-state basis. The American College of Obstetricians and Gynecologists recommends that medical schools include abortion training in their curriculum. It is still unknown if the Dobbs decision will limit medical students’ abortion training and/or discourage them from pursuing a career in obstetrics. The goal of this study was to investigate the attitudes of medical students toward abortion following the Dobbs decision and their beliefs about its possible effect on future medical education and practice.

Methods

This cross-sectional, observational study collected data from students enrolled in a Florida, United States osteopathic medical school using an anonymous online questionnaire from February to March 2023. The questionnaire contained 35 items that addressed medical students’ attitudes towards a range of potential implications on medical training due to the new abortion restrictions. Hypothesis testing was performed using Spearman's rho correlation and multivariate linear regression to explore the relationship between the predictor variables (concerns about future practice regarding abortion, religiosity, and acceptability of abortion based on gestation age) and the predicted variable (attitudes about abortion). Data were analyzed using Statistical Package for the Social Sciences (IBM SPSS Statistics for Windows, IBM Corp., Version 28.0, Armonk, NY).

Results

In total, 158 participants completed the questionnaire; 91 (57.6%) were women. The mean age was 25.8 (range 21-37 years). Using a stepwise regression analysis, only the variables shown to be statistically associated (per Spearman’s rho bivariate correlation) with the predicted variable (abortion attitudes) were entered into the model (i.e., concerns about abortion education and future practice, religiosity, and abortion acceptability based on gestational age). A significant regression equation was found (F(3,134) = 205.750, p < 0.001, R2 = 0.822, R2 adjusted = 0.818). The percentage of variance in the scores accounted for by the model was 82%. Higher levels of feeling that the abortion ban would negatively affect their training and future practice, greater religiosity, and acceptability of abortion at later gestation ages were statistically significant predictors of more positive attitudes toward abortion in this sample of osteopathic medical students.

Conclusions

The results suggest that the attitudes of medical students toward abortion are related to multiple factors, including concerns about future abortion training, religiosity, and the week of pregnancy acceptable for a woman to have an abortion. Findings also highlight the attitudes of medical students in response to more restrictive abortion legislation, emphasizing their desire for possible curricular enhancements to safeguard their training and education.

## Introduction

The Dobbs v. Jackson Women's Health Organization (2022), herein referred to as Dobbs, overturned Roe v. Wade, the landmark United States (U.S.) Supreme Court ruling that recognized a woman's right to have an abortion [1]. Consequently, states have introduced new regulations and bans on abortion procedures. For example, in Texas, the "Texas Heartbeat Act," which restricts abortions after six weeks of pregnancy and imposes significant financial penalties on those involved, was passed [2]. Following the Dobbs decision, Texas also enacted House Bill 1280, imposing penalties on providers performing or aiding abortions without considering cases of rape, incest, or the well-being of pregnant women [3]. Subsequently, more states implemented similar restrictive measures, severely limiting access to abortion with few exceptions [4]. Other states have followed suit and introduced bans based on gestational age, specifying the number of weeks during which abortions can be legally performed [5].

Religiosity and abortion: a complex intersection

Religion and religiosity may influence one’s attitudes toward abortion [6]. Research has shown that religiosity (i.e., the extent to which one participates in religious practices or customs), and religious affiliation can be a strong factor in predicting attitudes about abortion in medical students [7-9]. Medical students, not unlike physicians who identified as Catholic, Protestant, or Evangelical, generally opposed abortion, whereas students who identified as Jewish or non-religious were more likely to support abortion [8-11]. As with differing opinions regarding abortion in the general population, medical students’ attitudes toward abortion may differ based on religiosity regardless of religious affiliation. Previous literature on abortion and the influence of religion on attitudes toward it indicates religiosity, not religious affiliation, maybe a better predictor of abortion attitudes in the U.S. population and medical students as well [8]. Exploring these factors is important to understand the complexity of attitudes toward abortions among future physicians.

Fetal viability, legal, and moral concerns

Opponents of late-term abortions often raise concerns about fetal viability, contending that as the pregnancy progresses, the fetus gains the ability to survive outside the womb [12]. This viewpoint is rooted in ethical and moral considerations about the potential for fetal consciousness and the sanctity of life. Evidence exists discussing the increased likelihood of moral distress and ethical conflict about abortion post-Dobbs among medical students as the bans challenge medical ethics and the tenets of professionalism [13].

Several factors contribute to delays in getting an abortion [12]. Moreover, fetal viability is not set at a specific date in the pregnancy; multiple factors determine viability, including gestational age, fetal weight and sex, and available medical interventions [12]. Since Dobbs, more states have passed laws restricting access to abortions later in pregnancy, by putting gestational age limits on abortion, or banning clinicians from performing certain procedures [14]. As of June 2023, 11 states have pre-viability gestational bans [15]. Restrictions such as this limit access for women seeking abortions even within the limited gestational period [16].

Medical students’ attitudes about abortion

Abortion continues to be an ethically challenging topic within medical provision, and medical students' attitudes toward abortion play a crucial role in shaping the future of reproductive medicine. Although medical students are more supportive of abortion than students of other non-health-related disciplines, their feelings about abortions vary [17,18] and are based in part on the patient’s reasons for seeking an abortion [8]. As seen in the general population, some students believe in a woman’s right to an abortion under any circumstance, those that are adamantly against abortion, and students whose support for abortion is conditional [10] (i.e., abortion acceptable based on the circumstances, such as in the instance of rape or health risks) [8]. Osteopathic principles include approaching patients with a holistic eye including patient autonomy and respect for private life. However, while osteopathic and allopathic medicine have different philosophical approaches to patient care, their views on abortion may be diverse and still unknown if driven by personal beliefs rather than inherently determined by their medical training.

Impact on abortion practices after Dobbs

Medical institutions and professional organizations have increasingly advocated for the integration of abortion education into medical curricula. This shift reflects a broader acknowledgment of the importance of reproductive healthcare and the understanding that abortion is a legitimate and essential part of women's reproductive rights. Students in states with increased abortion restrictions may have less (or no) clinical exposure [19]. A committee opinion by the American College of Obstetrics and Gynecologists (ACOG) found that although abortion education improved, approximately 70% of students still considered their abortion training inadequate during their third-year rotation [19]. Students who have received clinical abortion education found it valuable and desire that it be integrated into the training curriculum [19].

There has been growing recognition of the need for standardized and comprehensive abortion training [20]. The ACOG had previously recommended that medical schools and obstetrics and gynecology (OB/GYN) residencies incorporate training on abortion practices and procedures [21]. However, some residency programs lack or restrict abortion education [22]. Due to post-Dobbs state laws on abortion, more than two-thirds of U.S. medical students may face disruptions in abortion education [22]. These restrictions have the potential to affect medical education and training and limit the knowledge and skills needed to provide quality care for women provided by future physicians [22]. For these reasons and other concerns, medical trainees may be compelled to travel domestically or internationally for family planning rotations, placing added financial, logistical, and personal burdens on them due to such requirements [23].

There is evidence to suggest that medical students’ intentions to provide abortions while in their undergraduate years are better predictors of future abortion provision than during residency [24]. Within this context, this study aimed to assess factors that might influence medical students' attitudes toward abortion, specifically concern about future training and practice, abortion acceptability based on gestational age, and other sociodemographic factors including religiosity. Medical student attitudes may contribute to the expansion and normalization of abortion procedures training and future practice [24]. Medical educators will need to continually monitor the impact of diminished exposure to training on abortion.

Research questions and hypothesis

This study aimed to explore the attitudes of medical students towards abortion following the Dobbs decision that motivated more restrictive abortion laws in states across the United States. Of particular interest was how these changes might influence their concerns about their future choices of medical practice involving abortion provision. The study sought to answer the following questions: (1) What are medical students' attitudes and perceptions about abortion since the Dobbs decision? and (2) Does concern about future practice options, fetal viability and moral concerns, and religiosity significantly influence medical students' attitudes towards abortion? It was hypothesized that greater concern for the future of medical practice involving abortion provision, acceptability of abortion in later stages of pregnancy, and lower levels of religiosity would predict more positive attitudes towards abortion in medical students.

## Materials and methods

Overview

Approval for this study was granted by the researchers’ university’s Institutional Review Board (IRB; protocol #2023-37). This cross-sectional, observational study used a quantitative questionnaire to assess the attitudes and opinions of Obstetrics and Gynecology medical students (OMS) about abortion following the Dobbs decision. Descriptive statistics, correlation analysis, and multiple linear regression analysis were performed using Statistical Package for the Social Sciences (IBM SPSS Statistics for Windows, IBM Corp., Version 28.0, Armonk, NY) [25].

Participants and recruitment

The questionnaire was sent via the researchers’ university’s student body email listserv to the 1,594 medical students enrolled in years 1 through 4. All participants consented to participate in the study per IRB guidelines. Data were collected from January 31 to March 9, 2023. Study data were collected and managed using Research Electronic Data Capture (REDCap; Vanderbilt University, Nashville, TN, USA) electronic data capture tools hosted at (researcher’s institution omitted for blind review) [26,27]. REDCap is a secure, web-based software platform designed to support data capture for research studies, providing (1) an intuitive interface for validated data capture; (2) audit trails for tracking data manipulation and export procedures; (3) automated export procedures for seamless data downloads to common statistical packages; and (4) procedures for data integration and interoperability with external sources. REDCap ensures anonymity by using a numerical coding protocol that does not store any identifying information. Email reminders were sent after a week, then again after two weeks, and ultimately one week before the close of the survey.

Assessment instrument

The assessment instrument is found in the Appendices. Published articles from peer-reviewed scientific journals informed the researchers in the development of the research questions and selecting the major study variables. After considering the context of those publications, major study variables were selected based on what was thought to be associated with medical students' attitudes about abortion post-Roe v. Wade. The final questionnaire contained 35 items that addressed medical students' attitudes towards a range of potential implications on medical training due to recent abortion restrictions across the U.S. Items were categorized under five domains: (1) medical students' attitudes on abortion (18 items), (2) implications of the Dobbs decision on future practice (seven items), (3) acceptability of abortion based on gestation age (one item), (4) religious identification and religiosity (two items), and (5) sociodemographic factors and other sample characteristics (seven items).

Abortion attitudes

The fully validated Abortion Attitude Scale was used in the 18-item measure with the modification of the first item, “The Supreme Court should strike down legal abortions in the United States” to “The Supreme Court’s decision to overturn Roe v. Wade in the Dobbs v. Jackson Women’s Health Organization case is correct” to make it timelier and more relevant to this study [28]. The 18-item measure used a six-point Likert response set (6=strongly agree, 5=agree, 4=slightly agree, 3=slightly disagree, 2=disagree, 1=strongly disagree, whereby several items were reverse scored). After the reversal of selected items, higher scores indicated more positive attitudes toward abortion. Examples of items in this scale included “Abortion is wrong no matter what the circumstances are,” “A fetus isn’t a person unless it can survive outside the mother’s body,” and "Abortion should be an available alternative for pregnant teenagers." The total estimate of the reliability (Cronbach's Alpha) of the Abortion Attitude Scale has been reported to be .92 [28].

Fetal viability and moral concerns

A single item developed by the researchers was included that asked the participants to express their opinion as to up to what number of weeks (choices were 1-40) is having an abortion acceptable (whereby “0” meant unacceptable at any week of pregnancy and 40 weeks being the upper limit). This item was added to investigate whether a general consensus among OMS exists in terms of acceptability based on gestational age.

Concerns about future abortion practice post-Dobbs

The researchers developed a set of seven questions based on the existing literature to assess medical students' attitudes about future practice in which abortion services would be rendered. Examples of the items included "I believe sharing my beliefs on abortion may prevent me from getting a job as a physician in the future" and "The criminalization of abortion will hinder my ability to care for my future patients." Participants were asked to respond to the items using a six-point Likert scale (6=strongly agree, 5=agree, 4=slightly agree, 3=slightly disagree, 2=disagree, 1=strongly disagree). Higher scores indicate greater concerns about the future of medical practice regarding abortion. The complete assessment instrument is found in the Appendices.

Religion and religiosity

Data were collected on participants’ self-reported religion by asking the question, “Regarding, religion/spiritual belief or practice, how do you identify” and offering a list of 10 common world religions with the additional choices “other,” and “none.” Religiosity was measured using one item (“How important is religion in your life?”) developed by the researchers using a four-point Likert-type response set (1=very important, 2=somewhat important, 3=not too important, 4=not important at all). Higher scores on this item indicate lower levels of religiosity.

Sample characteristics

Seven categorical items asked participants about their age, race, gender identification, sex (at birth), and year in the medical school program. Additional items asked participants if they had an abortion in the past (response options “yes,” “no,” “not applicable,” and “prefer not to answer”) and if someone close to them has had an abortion (“yes,” “no,” and “prefer not to answer”).

Participant response

During the data collection period, 218 participants returned the questionnaire (13.7% response rate). After excluding cases with less than 66% of the survey items answered 158 completed questionnaires were kept (72% completion rate) and used in the final analysis.

Pre-analysis

A post hoc power analysis was conducted to assess the statistical power of our multiple regression analysis, which included three predictors: (1) concern that the abortion ban would affect training and future practice, (2) religiosity, and (3) acceptability of abortion based on gestation age. The analysis was performed using G*Power software (Heinrich-Heine-Universität Düsseldorf, Düsseldorf, Germany). We used the observed effect size from the analysis, the sample size (N=158), and the alpha level (0.05) used in the study. The purpose was to evaluate the adequacy of statistical power in detecting significant effects given the observed data. The post hoc power analysis revealed that the achieved level of statistical power for our multiple regression analysis with three predictors was 0.988. This indicates that there was a 99% probability of detecting significant effects given the observed effect size (i.e., medium effect size), sample size, and alpha level used in the original analysis. The findings of the post hoc power analysis suggest that our study had adequate statistical power to detect significant effects of the three predictors on the outcome variable. However, it is essential to interpret the results cautiously, considering potential limitations such as the reliance on observed effect sizes and the retrospective nature of the analysis. The post hoc power analysis, however, provides valuable insights into the adequacy of statistical power in our study. The achieved power level of 0.988 indicates a high probability of detecting significant effects, enhancing confidence in the validity of our study findings.

The SPSS [25] was used for the analyses after downloading the data from REDCap [26,27]. Visual inspections of the observed distributions and tests for skewness and kurtosis (i.e., assessment for normal distributions) were performed. For this sample, reliability estimates (i.e., Cronbach’s alpha) for the two scaled measures (“abortion attitudes” and “concern for abortion training and future practice”) were computed (α = 0.936 and 0.841, respectively), both of which were within acceptable limits (α >.70) [29]. Discrete variables are reported as frequency and percentage. Continuous variables (e.g., age, acceptability of abortion based on gestational age) were reported as means and standard deviation (SD). Although there is support for treating Likert-type (ordinal) variables with five or more categories as continuous without any harm to the analysis, the six-point Likert-type scales used in this study were treated as ordinal and analyzed as such [30-33]. Therefore, the modes (and not means and standard deviations) were reported for the variables abortion attitudes, concern for future abortion education and practice, and religiosity. Spearman’s rho correlation and multivariate linear regression were used for hypothesis testing. Before performing the regression, multicollinearity testing was conducted. The independent variables were within acceptable variance inflation factors limits. Moreover, the scales were normally distributed (i.e., the data did not deviate from normality enough to affect inference).

## Results

Characteristics of the sample (n=158)

The mean age of the participants was 25.8 years (SD = 2.46; range 21-37 years). The majority (n=90; 57.3%) identified as female/woman; 63 (39.9%) identified as male/man; four (3.2%) declined to answer the item. Fifty-four (34%) were first-year students, 59 (37.3%) were second-year students, 26 (16.5%) were third-year students (in clinical rotations), and 19 (12%) were fourth-year students (in clinical rotations). Regarding race, the majority of the participants identified as white/Caucasian (n=116; 73.4%), 24 (15.2%) reported being Asian, and 18 (11.4%) preferred not to report their race. More than one-third (n=61; 38.6%) of the participants reported that someone close to them had an abortion, 84 (53.2%) reported they did not have someone close to them have an abortion, and nine (8.2%) preferred not to answer. Only three (1.9%) of the participants reported having had an abortion; the rest reported they had not had an abortion (n=97; 61.4%), not applicable (n=53; 33.5%), or preferred not to answer (n=5; 3.1%). Table 1 reports the religious/spiritual affiliation of the participants.

**Table 1:** Frequencies of Religious/Spiritual Belief or Affiliation (N=158)

Religious Beliefs by Denomination	Frequency (n)	Percent (%)
Christian - Denominational (e.g., Catholic, Protestant, Lutheran, Baptist, Orthodox)	57	36.1%
Atheist/Agnostic	26	16.5%
Christian (non-denominational)	20	12.7%
Jewish	11	7.0%
None	10	6.3%
Muslim	8	5.1%
Other	7	4.4%
Hindu	6	3.8%
Prefer not to answer	6	3.8%
Buddhist	3	1.9%
Sikh	2	1.3%
Latter-Day Saint	2	1.3%

Religiosity

**Figure 1:**
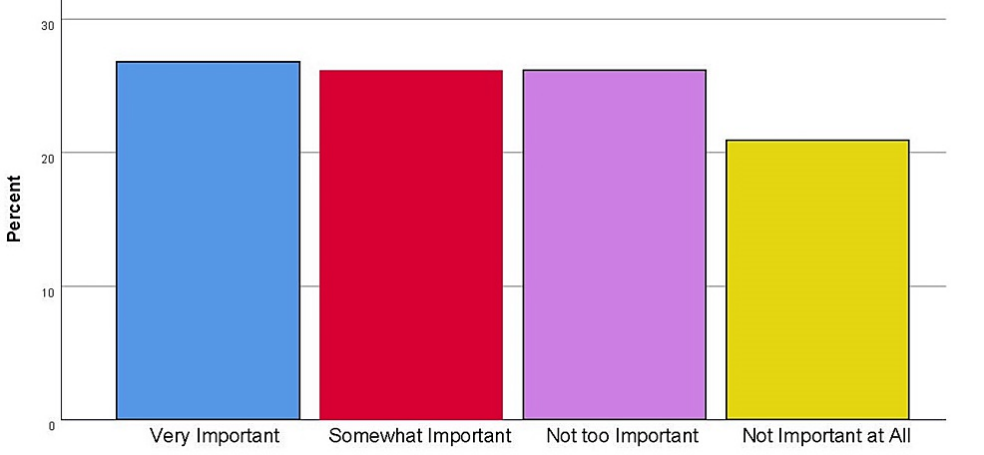
Religiosity Frequencies Participant responses to the item “How important is religion in your life” (i.e., religiosity) are reported by percent.

Participant responses to the item “How important is religion in your life” are reported in Figure 1. Forty (26%) of the participants reported that religion was very important in their life; 40 (25.3%) reported that it was somewhat important; 40 (25%) reported that it was not too important; and 32 (20.3%) reported that it was not important at all. Five (3.2%) of the participants declined to respond.

Acceptability of abortion based on gestational age

**Figure 2:**
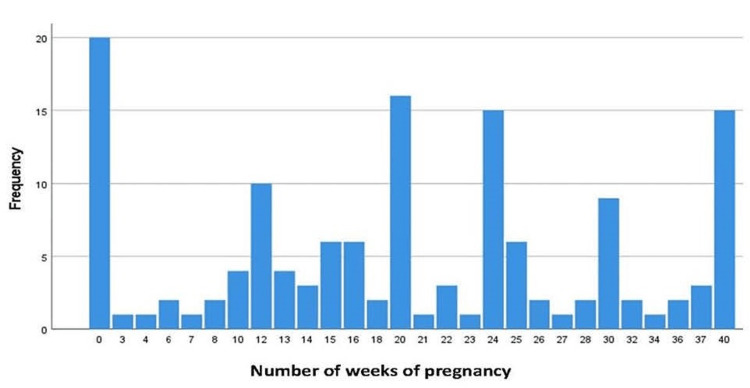
Acceptability of Abortion Based on Gestational Age Scoring: “0” weeks indicates abortion is not acceptable at all and "40" weeks indicates acceptability at any gestational age; weeks 1-39 indicate variability based on the exact week of acceptability.

Figure 2 reports the participant responses about gestational age and abortion. Twenty (12.7%) respondents reported “0” weeks, indicating abortion was not acceptable at all, and 15 (9.5%) participants reported abortion acceptability at 40 weeks (i.e., what would be considered full-term pregnancy). As seen in Figure 1, acceptability for abortion reported by the participants was variable, with notable peaks at 12 weeks, 20 weeks, 24 weeks, and 30 weeks.

Summary statistics for the major study variables

Table 2 reports the summary statistics for the major study variables. The mean score for the item that measured the acceptability of abortion based on gestational age was 19.46 (SD=12.065), with higher scores indicating more acceptability for abortion at later gestational ages. The mode for the scale that measured attitudes toward abortion (18 items using a six-point Likert response set) was 5, with higher scores indicating more positive attitudes toward abortion. The mode for religiosity was 1 (single item with a four-point Likert response set), with higher scores indicating less importance of religion in their life. The mode for the measure that assesses concerns about abortion education and future practice (seven items using a six-point response set) was 5, the smallest reported among multiple modes for that scale. Higher scores on the concerns scale indicate more concern about the lack of abortion education and future clinical practice regarding abortion.

**Table 2:** Summary Statistics for the Major Study Variables ^a^ SD indicates the standard deviation. ^b ^Range indicates the Likert-type scale score range for each item of the scale.

Major Study Variable	n	Mean	Mode	SD^a^	Range^b^
Acceptability of abortion based on gestational age	141	19.46	0	12.065	0-40
Religiosity	153	N/A	1	N/A	1-4
Concerns about abortion education and future practice	158	N/A	5	N/A	1-6
Attitudes about abortion	158	n/A	5	N/A	1-6

Correlations

Table 3 reports the statistically significant Spearman’s rho correlations between the major study variables and the predicted variable (abortion attitudes). None of the demographic variables (e.g., race, age) were correlated to the predicted variable. Only those predictor variables that were statistically significantly correlated with the predicted variable were entered into the regression model.

**Table 3:** Correlation of Major Study Variables With Abortion Attitudes * Correlation is significant at the 0.01 level (two-tailed).

Major Study Variable	Abortion Attitudes
Concerns about abortion education and future practice	0.707^*^
Gestational age	0.704^*^
Religiosity	0.480^*^

Multiple regression

Table 4 reports the multivariate linear regression analysis that used a stepwise process where all the predictors enter the regression together. Only the variables shown to be correlated (using Spearman’s rho bivariate correlation) with the predicted variable (abortion attitudes) were entered into the model (i.e., concerns about abortion education and future practice, religiosity, and abortion acceptability based on gestational age). None of the demographic variables were correlated with the outcome variable and were thus not entered into the model. Multicollinearity tests were performed, and variance inflation factors were within range. A significant regression equation was found (F(3,134) = 205.750, p < 0.001, R2 = 0.822, R2 adjusted = 0.818). The regression coefficients for the predictor variables are as follows: concerns about abortion education and future practice (1.158), religiosity (3.621), and abortion acceptability based on gestational age (0.549). Collinearity statistics, standard errors, t-values, and p-values to indicate statistical significance are included in Table 4. The percentage of variance in the scores accounted for by the model was 82%. Higher levels of feeling that the abortion ban would negatively affect their training and future practice, greater religiosity, and acceptability of abortion at later gestation ages were statistically significant predictors of more positive attitudes toward abortion in this sample of medical students.

**Table 4:** Regression Model of Predicted Variable “Abortion Attitudes” Predicted variable: abortion attitudes *p < 0.001.

Variables	B	Std. Error	Beta (β)	t	Sig.	Collinearity Statistics	
Predictors	N/A	N/A	N/A	N/A	N/A	Tolerance	VIF
(Constant)	23.267	2.685	N/A	8.666	<0.001*	N/A	N/A
Concerns about abortion education and future practice	1.158	0.109	0.520	10.657	<0.001*	0.560	1.787
Religiosity	3.621	0.689	0.213	5.259	<0.001*	0.809	1.235
Abortion acceptability based on gestational age	0.549	0.077	0.350	7.087	<0.001*	0.544	1.837

## Discussion

This study provided some insight into several factors that might influence medical students' attitudes toward abortion, particularly related to fetal viability and moral concerns, the importance of religion in their lives, and their concerns about abortion training and future practice.

Acceptability of abortion based on gestational age

Significant associations were found between participants’ acceptability of abortion based on gestational age and their attitudes about abortion. Previous research has indicated that although medical students may be more supportive of abortion than students of non-medical fields, their attitudes about abortions can vary [17,18] and may be dependent on the woman’s reasons for wanting an abortion [8,10]. This may have something to do with the acceptability of abortion at later gestation ages predicting more positive attitudes toward abortion. Interestingly, there were opposing “peaks” (e.g., high numbers of responses) about acceptable gestational age for terminating a pregnancy (regardless of the reason for termination). The first peak, occurring at 12 weeks (the end of the first trimester) was in contrast to the second peak, occurring at 20 weeks (at which time an anatomy scan is conducted to examine a fetus’ organs). The third peak, at 24 weeks, relates to the earlier legal limit mandated by Roe v. Wade. Medical students who have completed their clinical coursework are generally familiar with these stages, which may have influenced their attitudes about which week it is acceptable to end an unwanted pregnancy. Attitudes about gestational age and abortion acceptability need further investigation with larger samples in different states in the United States to tease out influences based on demographic, legal, and other regional differences.

Navigating future reproductive health practice challenges

Another important aspect that is just being explored in the literature is medical students’ concern for abortion education and training coupled with their uncertainty about future clinical practice in a post-Roe v. Wade era. While it may be too soon to ascertain the downstream effects of the Dobbs decision, preliminary findings from this study suggest some trepidation going forward regarding choosing an OB/GYN specialty and whether will they be adequately trained in medical school.

The finding that students in this sample reported concern about the future of abortion training in undergraduate and graduate training is not surprising, as earlier literature states that some residency programs already lack or restrict abortion education and, due to the reversal of Roe v. Wade, medical students may face an even more diminished version of abortion training [22]. Recent reports indicate that medical students are already seeking training overseas for family planning rotations [23]. As we look to the future, the landscape of medical education is likely to evolve in response to the ongoing discourse surrounding reproductive health and abortion. For this reason, this study sought to assess OMS’ concern for how it may affect their future intentions regarding medical practice in the area of reproductive health.

Religiosity

Religiosity has been known to play a role in shaping societal norms and influencing the legal landscape regarding abortion. This study thus sought to explore the contribution of religiosity to attitudes toward abortion in a sample of medical students. Findings from the regression modeling indicated that religiosity significantly influenced personal views on abortion and influenced their stance on the permissibility of abortion.

Religious affiliation in this sample was varied. Findings from this study found that lower levels of religiosity predicted more positive attitudes toward abortion (in general), findings which are supported by earlier literature [6-9]. About half of the participants reported religion was very important or somewhat important; the other half reported that it was not too important or not important at all (five participants declined to answer).

The relationship between religiosity and abortion is intricate and multifaceted, reflecting the diversity within religious traditions and the nuanced ways in which individuals interpret and apply religious teachings to their beliefs. Medical students will need to navigate the complexities of abortion debates in addition to its variable legality based on region. Exploring the role of religiosity may be important to understanding the complexity of attitudes toward abortions among future physicians.

Abortion attitudes

It was hypothesized that higher levels of concern that the abortion ban would negatively affect their training and future practice, greater religiosity, and acceptability of abortion at later gestation ages would predict more positive attitudes about abortion. The model for the abortion attitudes scale was high (5 within a Likert scale range of 1-6, with higher scores indicating more positive attitudes toward abortion).

This finding is supported by previous literature indicating that medical students overall hold positive attitudes toward abortion [17,18]. However, it is interesting to note that even though attitudes about abortion were favorable in this sample of medical students, responses to acceptable gestational age for abortion varied greatly. One measure for attitudes toward abortion with general terms about abortion may not capture all the nuance of this deeply intimate and highly complicated issue. More research may be warranted to ascertain to what extent fetal viability concerns and worries about adequate training and future clinical practice drive medical students’ attitudes about abortion in diverse contexts.

Some of the participants in this study began medical school before the Dobbs decision, and it might be of value to see if some students will change their attitude toward abortion since the new law. As the majority of these women students are of child-bearing age, it might be worth investigating their attitudes about abortion vis a vis their personal lives as well as their professional ones.

Implications for osteopathic medical education

As medical technology advances, the definition of viability becomes a pivotal point in the abortion discourse, prompting discussions about when the state's interest in protecting potential life should override a woman's right to choose [12]. The controversy surrounding the timing of abortion underscores the intricate interplay between individual rights, moral considerations, and societal values [34]. The key perspective is encouraging health providers to recognize the nuances, appreciate diverse perspectives, and work towards a compassionate and informed understanding of abortion and its timing. In turn, these attitudes might affect abortion and education training in medical schools.

Abortion training is crucial for several reasons. First, it ensures that healthcare professionals have the necessary clinical skills to perform abortions safely, minimizing potential risks to the patient's health. Second, it helps providers develop the sensitivity and empathy needed to offer non-judgmental and supportive care to women facing unplanned pregnancies.

Abortion is a common medical procedure, and healthcare professionals must be adequately trained to provide safe and compassionate care to women seeking these services. Proper training encompasses the technical skills needed for different abortion methods, such as medication abortion, aspiration (surgical) abortion, and dilation and evacuation. It also includes knowledge about potential complications, side effects, and the ability to manage emergencies effectively. Abortion training encompasses a wide range of skills, including medical, ethical, and communication skills, to ensure that healthcare providers can navigate the complexities associated with abortion care. However, students in states with increased abortion restrictions may have less (or no) clinical exposure [19].

The tenet of osteopathic medicine is grounded in the philosophy that posits rational treatment is based on, among other factors, the ability of patients to understand and manage their behavior as well as the reactions to one’s feelings and environmental influences. Osteopathic principles also include looking at patients with a holistic eye and becoming familiar with each patient as a whole person - mind, body, and soul. This lends itself to respect for patient autonomy, the right to dignity, and respect for private life without compromising the highest attainable standard of health care. For some medical students, more restrictive laws on abortion may create moral distress later when they begin graduate training and face the abortion restrictions firsthand. While osteopathic and allopathic medicine have different philosophical approaches to patient care, their views on abortion are diverse and driven by personal beliefs rather than inherently determined by their medical training.

It may be important to integrate not only abortion training on practice and procedure, but also how to navigate the personal, ethical, and legal waters as they strive to do their best for their patients in the current political climate. Future physicians should be aware of legislative changes, advocacy strategies, and the impact of policy decisions on patient access to reproductive services. Osteopathic medical education programs will need to address healthcare disparities related to access to abortion and other reproductive services. Training should emphasize the importance of mitigating barriers to care, including geographic, economic, and social factors, to ensure equitable access for all women.

Directions for future research

Several areas exist for future research to explore to enhance abortion education in medical school. First, comprehensive evaluation studies of current abortion training programs to determine their effectiveness in preparing students for providing comprehensive abortion services are needed. This can involve assessing students' knowledge, skills, and attitudes before and after training. Second, longitudinal studies could be conducted to track medical students' attitudes about abortion throughout their medical education and into their clinical practice. This could provide insights into the long-term impact of training on students' attitudes and willingness to provide abortion care. Third, studies that assess the effectiveness of incorporating experiential learning methods, such as simulation-based training, into abortion education could prove useful. Simulation can provide students with realistic clinical scenarios to practice abortion procedures in a safe and supportive environment. Last, research is needed to explore strategies for overcoming potential barriers to implementing comprehensive abortion training in medical schools, such as institutional policies, faculty attitudes, and lack of resources. and promoting a supportive learning environment for students. By addressing these research areas, medical educators may be better positioned to prepare future physicians by integrating comprehensive, patient-centered abortion care into their curricula.

Limitations of the study

Several limitations of this study should be noted. First, through convenience sampling recruiting methods, the true distribution of the osteopathic or allopathic medical student population could not be gauged. Because the participants were from a Florida-based osteopathic medical school, medical students from other regions were not fully represented. Also, due to the nature of online surveys (low response and low completion rates) and the brief data collection window sent to busy medical students, the resulting sample size was relatively small. It is important to note that the study was conducted in Florida, a state with stringent abortion laws, which could have influenced how participants responded to the survey. Furthermore, it cannot be determined whether non-osteopathic medical students would have different responses to the survey items than allopathic medical students. Moreover, the study was voluntary and may have an increased risk of sampling bias since students more likely to complete the survey may have a greater interest in abortion (in favor or against). Cause-and-effect relationships or changes over time cannot be predicted using a cross-sectional survey design such as the one used in this study. Last, while the achieved power was high, a larger sample size would further strengthen the generalizability of the findings. Given the aforementioned limitations, results generated from this study should be taken with caution.

## Conclusions

The results of this study show that OMS with more concern for future clinical practice options, less religiosity, and more acceptance of abortion at a later gestational age have more positive attitudes about abortion in general. An interesting finding showed that although most OMS in this sample had favorable attitudes toward abortion, the gestational age at which abortion should be legal varied greatly. Not surprisingly, those OMS with lower levels of religiosity held more favorable attitudes about abortion. These results add to other bodies of literature regarding attitudes about abortion attitudes in medical students, further showing more support for continuing exploration as the political climate and abortion laws continue to change. More research is necessary to further explore medical trainees’ attitudes toward abortion as new restrictions on abortion continue to emerge. As medical education evolves, ongoing efforts should be made to provide comprehensive and unbiased reproductive health education that prepares future osteopathic physicians to navigate the ethical and clinical challenges associated with abortion.

## Figures and Tables

**Figure 3:**
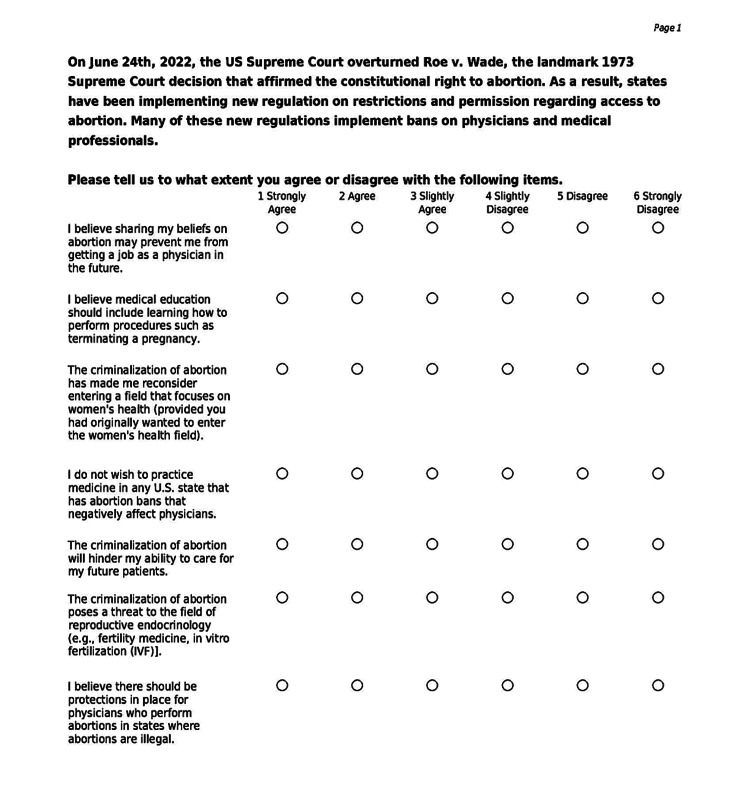
Survey page 1 Items 2, 5, 6, 8, 11, 13, 15, 16, and 17 are reverse scored, whereby 1=strongly agree, 2=agree, 3=slightly agree, 4=slightly disagree, 5=disagree, 6=strongly disagree.

**Figure 4:**
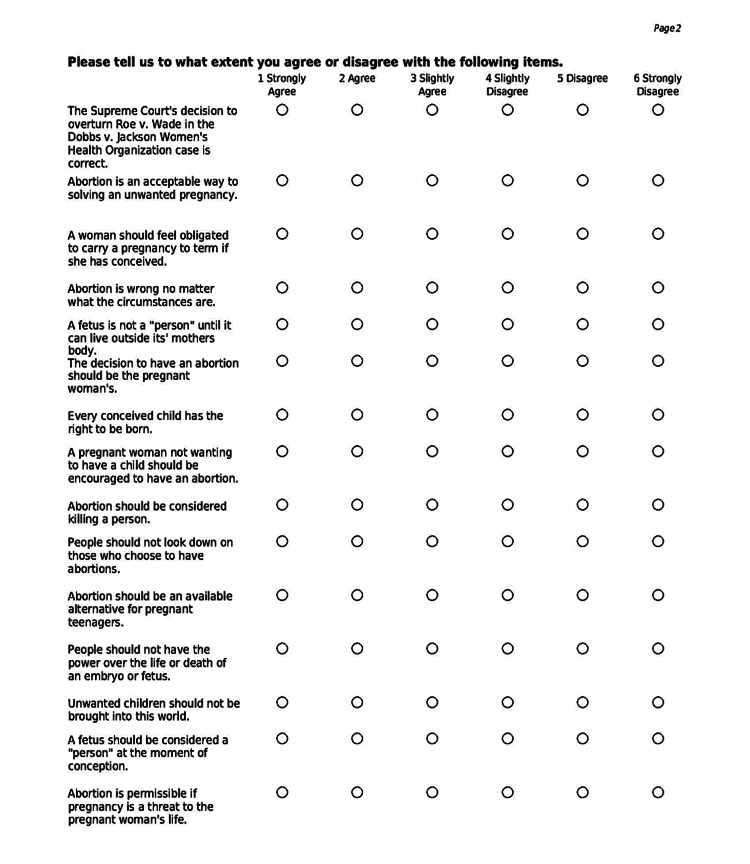
Survey page 2 Items 2, 5, 6, 8, 11, 13, 15, 16, and 17 are reverse scored, whereby 1=strongly agree, 2=agree, 3=slightly agree, 4=slightly disagree, 5=disagree, 6=strongly disagree.

**Figure 5:**
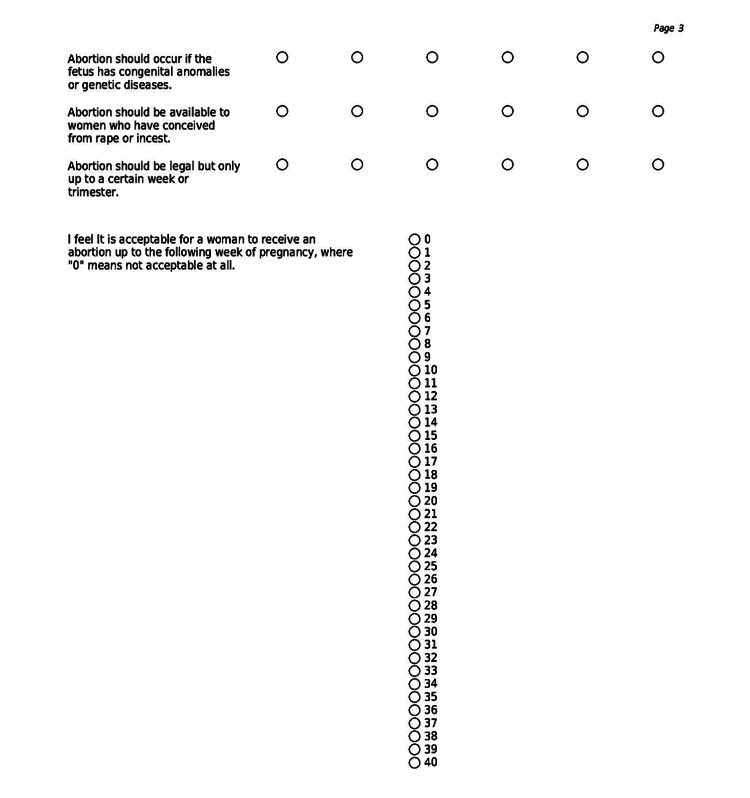
Survey page 3

**Figure 6:**
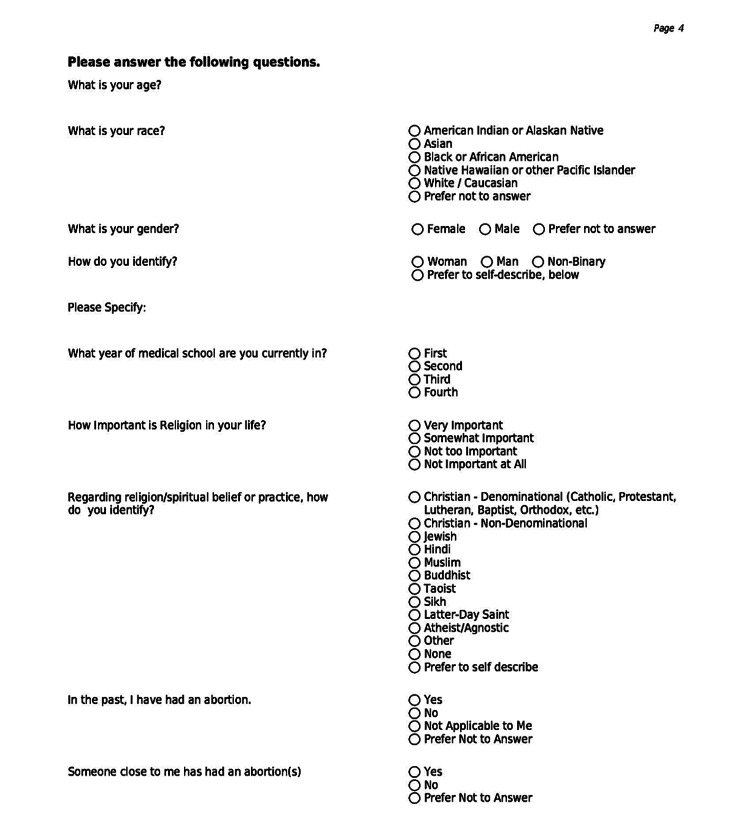
Survey page 4
